# Compliance of mothers following recommendations to breastfeed or withhold breast milk during rotavirus vaccination in North India: a randomized clinical trial

**DOI:** 10.1186/1745-6215-15-256

**Published:** 2014-06-28

**Authors:** Temsunaro Rongsen-Chandola, Brita Askeland Winje, Nidhi Goyal, Sudeep Singh Rathore, Madhu Mahesh, Rajat Ranjan, Alok Arya, Farhana Afzal Rafiqi, Nita Bhandari, Tor A Strand

**Affiliations:** 1 Centre for Health Research and Development, Society for Applied Studies, 45, Kalu Sarai, New Delhi, India; 2 Division of Infectious Disease Control, Norwegian Institute of Public Health, Oslo, Norway; 3 Innlandet Hospital Trust, Lillehammer, Norway and Centre for International Health, University of Bergen, Bergen, Norway

**Keywords:** rotavirus, Rotarix®, withhold breastfeeding, encourage breastfeeding, perception

## Abstract

**Background:**

Neutralizing antibodies in breast milk may adversely influence the immune response to live oral vaccines. Withholding breastfeeding around the time of vaccine administration has been suggested for improving vaccine performance. However, we do not know whether mothers find withholding breastfeeding around the time of vaccination acceptable and how they perceive this recommendation.

**Methods:**

In a clinical study designed to examine predictors of poor immune response to rotavirus vaccine in infants in India, Rotarix® was administered to infants at 6 and 10 weeks with other childhood vaccines. For the study, 400 mother–infant pairs were randomized into two groups in a 1:1 ratio. Mothers were either recommended to withhold breastfeeding or were encouraged to breastfeed half an hour before and after administration of Rotarix®. The mother–infant pairs were observed and the breastfeeding intervals were recorded during this period. Mothers were administered a questionnaire about their perception of the intervention after the infants received the second dose of Rotarix®.

**Results:**

Almost 98% (391/400) of the infants received both doses of Rotarix®. Adherence to the recommendations was high in both groups. All mothers in the group who were asked to withhold breastfeeding did so, except one who breastfed her infant before the recommended time after the first dose of Rotarix®. Of the mothers, 4% (7/195) reported that the recommendation to withhold breastfeeding was difficult to follow. All mothers in this group reported that they would withhold breastfeeding at the time of vaccination if they were asked to by a health-care provider. Only one mother responded that withholding breastfeeding would be a reason for not giving rotavirus vaccine to her infant.

**Conclusions:**

Withholding breastfeeding half an hour before and after vaccination appears to be acceptable to mothers in this setting. If withholding breastfeeding produces an improvement in the performance of the vaccine, it could be used to increase the public health impact of rotavirus immunization.

**Trial registration:**

Clinical Trial Registry, India (CTRI/2012/10/003057), Clinicaltrials.gov (NCT01700127).

Date of Registration: Clinical Trial Registry, India: 28 September 2012, Clinicaltrials.gov: 3 October 2012.

## Background

Rotavirus is the most common cause of severe dehydrating diarrhea worldwide in infants and young children, killing approximately 453,000 children under the age of 5 each year [1]. Rotavirus is particularly threatening in India, causing around 100,000 deaths in young children every year [2]. Vaccination remains a cornerstone in the prevention of rotavirus-associated morbidity and mortality.

The two oral rotavirus vaccines commercially available, Rotarix® (GlaxoSmithKline Biologicals, Rixensart, Belgium) and RotaTeq (Merck and Co, PA, USA), have been shown to be safe and effective [3]. Overall, rotavirus vaccines are associated with 74% and 61% reductions in very severe and severe rotavirus infections, respectively, and a 47% reduction in rotavirus-related hospital admissions [4-8]. However, in low-income countries, these vaccines have a lower efficacy compared to other oral vaccines like polio and cholera [9]. In impoverished high-mortality settings, host factors, including maternal antibodies, interfering bacterial and viral agents, and child and maternal malnutrition, may affect immune responses [10].

*In vitro* studies of the neutralizing effect of breast milk have suggested that withholding breastfeeding around the time of rotavirus vaccine administration may impact vaccine performance [11]. Efficacy trials have reported no difference between breastfed and non-breastfed infants. However, in these studies the breastfeeding practices were self-reported and the interval between breastfeeding and vaccine administration was not adequately evaluated [10,12,13]. Breast milk from Indian mothers is reported to have much higher concentrations of rotavirus-neutralizing antibodies than breast milk from mothers in industrialized countries [11]. In phase I/II studies of a recently developed rotavirus vaccine (116E) in India, breast milk was withheld for half an hour before and after each vaccine dose, and the seroconversion rate was almost 90% [14].

Other studies are also being conducted to assess the modifying effect of breast milk on vaccine efficacy [15,16]. If these studies demonstrate an improvement in the immune response by withholding breastfeeding around the time of vaccination, this practice could be used to increase the public health impact of rotavirus vaccination. However, little is known about mothers’ perceptions of withholding breastfeeding and whether such an intervention would be feasible and acceptable to mothers in low-income settings.

The primary aim of the study was to assess the impact of withholding breastfeeding compared to encouraging breastfeeding on the immune response to Rotarix® in infants. The results of the primary aim are not presented here. This paper describes the study methodology and the mothers’ ability to adhere to the breastfeeding recommendation as well as their perception of the recommendation.

## Methods

### Study setting

The trial was conducted in the urban resettlement neighborhoods of Govindpuri-Tigri-Dakshipuri, Tuglakabad and Sangam Vihar in South Delhi, India. These areas are typical urban resettlement neighborhoods.

### Randomization

The randomization list was generated by a statistician independent of the study team. Subject ID allocation for each participant was through serially numbered, opaque sealed envelopes.

### Sample size

The sample size was based on the primary aim of the study. Assuming 60% seroconversion in the infants whose mothers were encouraged to breastfed and 80% in the group in whom breastfeeding was withheld, at 90% power and alpha level of 5%, 200 infants were required in each group. This sample also accounted for 30% dropouts and 10% who might be excluded from the analysis because of high levels of antibodies at baseline.

### Enrollment and intervention delivery

Participants were enrolled into the study from October 2012. Infants aged less than 7 weeks were identified through a household survey. The families of infants aged 6 to 7 weeks were called to the study clinic for screening and enrollment. Infants were enrolled if their parents gave consent for participation, were aged 6 to 7 weeks, had a weight-for-age *Z* score that was not ≤ −3 [17] and were from a family with no plans to move out of the study area in the next 4 months. Infants were excluded if they were not breastfed, had already received a rotavirus vaccination, had a chronic enteric disease and illness requiring hospital referral or causing diarrhea on the day of enrollment or had a condition that the investigator judged to warrant exclusion, or if the mother or infant had an immune deficiency disease.

During consent, families were informed that if they agreed to take part in the study, their baby would be randomly selected for either the withhold breastfeeding or encourage breastfeeding group. All information was provided in the local dialect. The verbatim information in the informed consent form regarding allocation to either group was:

You are being asked permission for your baby to be screened for this study. This is to check if your baby is healthy enough to receive the rotavirus vaccine and the childhood vaccines. If your baby is assessed to be well enough to receive the vaccine, your baby can take part in this study. In case you agree to allow your baby to participate, your baby will be randomly (like tossing a coin) selected to either receive breastfeed or not be breastfed 30 minutes before and after receiving the Rotarix®.

It was also explained that the purpose of the study was to test: “The effect of not giving and giving breast milk on the antibody response of the two doses of Rotarix®”. The rationale for asking mothers to withhold or encourage breastfeeding was: “Your baby’s participation in this study may help in generating information about the usefulness of giving or not giving breast milk before and after the Rotarix® vaccine”.

After obtaining written informed consent, 400 eligible mother–infant pairs were enrolled and randomized into one of the two study groups.

Group 1: Mothers were advised to withhold breastfeeding 30 min before and 30 min after each dose of the vaccine.

Group 2: Mothers were encouraged to breastfeed immediately before and after each dose of the vaccine.

The recommendation was given by a trained study team nutritionist in both groups. The Group 1 mothers were told: “You have been selected to be in the group where breastfeeding needs to be withheld. Do not breastfeed your child for half an hour before and after receiving the rotavirus vaccine”. Group 2 mothers were told: “You have been selected to be in the group where you are encouraged to breastfeed your child in the half an hour duration before and after receiving the rotavirus vaccine”. At the study clinic there were two separate designated areas for the two groups. Each area was supervised by clinical coordinators.

Mother–infant pairs were required to wait in the designated area as per their group allocation. All activities, including specimen collection and administration of vaccines, were conducted in these areas. The team members were present in the same area to observe the mother–infant pairs during this time. After the 30 min of observation following Rotarix® administration, the infants were administered the other childhood vaccines. In line with usual practice, infants remained in the study clinic for another 30 min after administration of the childhood vaccines to allow observation, management and documentation of any immediate adverse events. In this observation period, the women were not given any specific breastfeeding instructions, although breastfeeding practices were recorded by the project team members.

Each enrolled infant was given two doses of the Rotarix® vaccine along with a pentavalent vaccine (diphtheria, pertussis, tetanus, hepatitis B and *Haemophilus influenzae*) and an oral polio vaccine. The vaccines were administered at the ages of 6 to 7 and 10 to 14 weeks, maintaining a minimum interval of 4 weeks between the two doses. The third dose of the pentavalent vaccine was offered to all infants when they came to the study clinic for the end of study activities 4 weeks after the second dose of Rotarix®.

After enrollment, participants were contacted weekly after each dose of Rotarix® to ascertain whether there were any signs or symptoms of suspected intussusception or whether they had suffered from an illness requiring hospital referral or had been hospitalized. Severe adverse events were reported to the Society for Applied Studies, Ethics Review Committee (SAS-ERC). The follow-up of the last child was completed in May 2013.

### Data collection

Baseline information on maternal and infant characteristics was collected at the time of enrollment. During the observation period, details, such as the time the observation started and ended and the duration of any breastfeeding, were documented in a form. After an infant received the second dose of Rotarix®, its mother was asked a set of structured questions about how she perceived the intervention (the recommendation to withhold or encourage breastfeeding). Mothers were also prompted to comment on their answers.

Biological specimens from mothers and infants were collected to assess immunogenicity. Baseline maternal blood and breast milk specimens, and infant blood, saliva and stool specimens were obtained. Before the second dose of Rotarix®, maternal breast milk specimens and infant saliva specimens were obtained. Four weeks after the second dose of Rotarix®, blood, saliva and stool specimens were collected from the infants.

### Presentation of data

Descriptive measures of continuous variables are presented as means and standard deviations (SDs) for symmetrical data, and as medians and interquartile ranges for skewed data. Descriptive measures of categorical data are presented as frequencies and percentages. An independent-samples *t*-test was used to explore the relationship between continuous variables.

### Ethical clearance

Ethical clearance was obtained from SAS-ERC (SAS ERC/43/2012) and the South-East Regional Ethical Committee of Norway (2012/193/REK). This study was conducted in compliance with the protocol Good Clinical Practices and other relevant regulatory guidelines.

## Results

**Figure 1:**
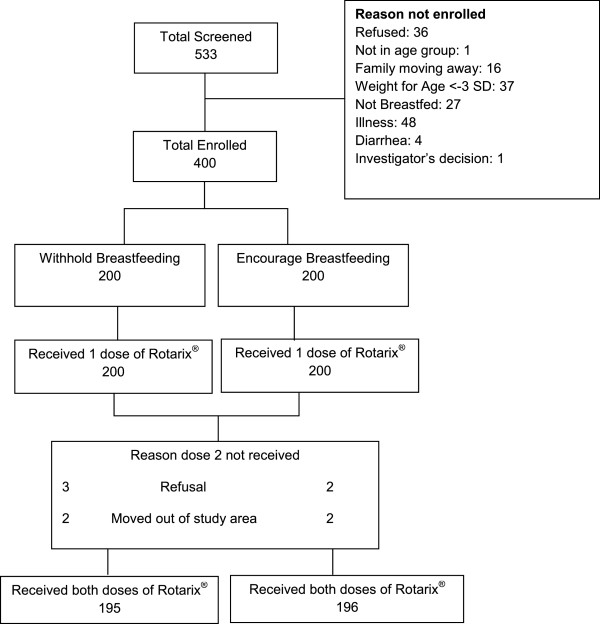
Trial profile.

Of the 533 infants screened for eligibility, 400 were enrolled and randomized (Figure 1). Baseline infant characteristics and socio-economic factors were comparable between the two groups (Table 1). Nine subjects did not receive the second dose of Rotarix®; five refused further participation and four moved out of the study area, leaving 391 subjects who received both doses of Rotarix® and whose mothers completed the questionnaire on their perception of the intervention.

**Table 1:** Baseline characteristics of participants in the study

	**Breastfeeding withheld (*n =*** **200)**	**Breastfeeding encouraged (*n =*** **200)**
**Infant characteristics**		
Age at enrollment (days) (mean and SD)	48 (4.0)	49 (3.8)
Birth weight* (kg) (mean and SD)	2.80 (0.4)	2.84 (0.5)
Weight at screening (kg) (mean and SD)	4.41 (0.6)	4.43 (0.5)
Sex:		
Boys	103 (51.5)	105 (52.5)
Girls	97 (48.5)	95 (47.5)
Exclusively breastfed	150 (75.0)	160 (80.0)
**Socioeconomic characteristics**		
Home birth	61 (30.5)	52 (26.0)
Type of family:		
Nuclear	112 (56.0)	115 (57.5)
Joint	88 (44.0)	85 (42.5)
Number of siblings (mean and SD)	0.95 (0.96)	1.1 (1.1)
Maternal age (years) (mean and SD)	24.4 (3.5)	24.8 (3.9)
Mother has not attended school	48 (24.0)	45 (22.5)
Father has not attended school	22 (11.0)	22 (11.0)
Family owns color television, cooler or scooter	182 (91.0)	179 (89.5)
Annual family income (rupees) (median and interquartile range)	84,000 (60,000, 120,000)	84,000 (72,000, 120,000)

All values are *n* (%) except when otherwise indicated. SD, standard deviation.

*Information on birth weight was available for 137 (68.5%) infants in the withholding breastfeeding group and 143 (71.5%) infants in the group encouraged to breastfeed.

The number of years in school varied greatly among both mothers and fathers, from 0 to 17 and 0 to 19, respectively. These distributions were similar in the two study groups. Mothers in the group withholding breastfeeding had a mean of 7.2 (SD, 4.8) years of education and those in the group encouraged to breastfeed had a mean of 7.0 (SD, 4.9) years. Similar figures were obtained for men (8.9 (SD, 4.1) and 9.1 (SD, 4.4) years, respectively).

With the exception of one mother, all mothers who were advised to withhold breastfeeding adhered fully to this recommendation (Table 2). Similarly, all mothers who were encouraged to breastfeed, except one, breastfed at least once (range one to four times) in each of the periods before and after vaccine administration. One woman did not breastfeed following the second dose of Rotarix®, but breastfed in the period before vaccine administration.

**Table 2:** Adherence to breastfeeding recommendations in the two groups

	**Breastfeeding withheld**	**Breastfeeding encouraged**
**Dose 1**	*n* = 200	*n* = 200
Time since last breastfeed (min) (median and IQR)	39 (33, 57)	100 (45, 152)
**Pre-vaccine observation**		
Number breastfed during observation period	0	200
Total breastfeed duration (min) during observation period (mean and SD)	−	11.8 (3.4)
**Post-vaccine observation**		
Number breastfed during observation period	1	200
Total breastfeed duration (min) during observation period (mean and SD)	0.01 (0.1)	7.7 (3.6)
**Dose 2**	*n* = 195	*n* = 196
Time since last breastfeed (min) (median and IQR)	56 (34, 70)	68 (43, 105)
**Pre-vaccine observation**		
Number breastfed during observation period	0	196
Total breastfeed duration (min) during observation period (mean and SD)	−	10.7 (2.8)
**Post-vaccine observation**		
Number breastfed during observation period	0	195
Total breastfeed duration (min) during observation period (mean and SD)	−	6.5 (2.4)

All values are *n* (%) except when otherwise indicated. IQR, interquartile range; SD, standard deviation.

The interval between the last breastfeed and the beginning of the intervention period varied widely among subjects, with maximum times of 491 and 388 min and minimum times of 6 and 9 min for the first and second doses, respectively. Mean intervals were significantly longer for infants who were given supplementary nutrition compared with those who were breastfed exclusively (mean difference, 40 min; *P* = 0.002).

Almost 78% of the infants were not given any other foods and fluids except breast milk; the mean number of breastfeeds per day was ten times. It was found that 75% of the infants in the group withholding breastfeeding were being exclusively breastfed. Infants were not breastfed for about an average of 49 and 46 min after receiving the first and second doses of Rotarix®, respectively.

Adherence to the breastfeeding recommendations was high in both groups. Half of the mothers in each group made additional comments on how they perceived the intervention. The main emerging theme in both groups was that the mothers found withholding breastfeeding around the time of vaccination acceptable and feasible, understanding that the vaccines were important and beneficial for their child and by withholding breastfeeding they could potentially improve the vaccine effect (Table 3).

**Table 3:** Mothers’ perception of breastfeeding recommendations

	**Yes**	**No**
**Breastfeeding withheld (*n =*** **195)**		
Found it difficult to withhold breastfeeding for 30 min before and after Rotarix®	7	188
She would withhold breastfeeding around time of vaccination if health-care provider asked her	195	0
Withholding breastfeeding would be a reason not to give rotavirus vaccine to her baby	1	194
**Breastfeeding encouraged (*n*** **= 196)**		
Found it difficult to breastfeed for 30 min before and after Rotarix®	1	195
She would breastfeed around time of vaccination if health-care provider asked her	196	0
Breastfeeding would be a reason not to give rotavirus vaccine to her baby	2	194

Among mothers who were asked to withhold breastfeeding for 30 min before and after vaccine administration, seven (4%) reported that this practice was difficult; five found withholding breastfeeding stressful when their infants cried, one found the interval to be too long and one made no comment. Three of these seven infants were breastfed exclusively. Only one mother reported that withholding breastfeeding would be a reason for not giving the rotavirus vaccine to her infant, commenting that the duration of the non-breastfeeding period was too long.

All mothers withholding breastfeeding reported that they would adhere to this practice if asked to do so by health-care professionals. Thirteen of them said that they would do so since they understood the importance of vaccination. Two mothers said that they did the same when the health workers in the immunization center asked them to do so during oral polio vaccine administration.

Of the mothers who did not find withholding breastfeeding difficult, nine commented that their baby was calm and slept during the observation period, eight commented that the observation period was not too long and five reiterated that they did not find it difficult to withhold breastfeeding.

## Discussion

This study assessed the feasibility of asking mothers to withhold breastfeeding. Mothers did not have any difficulty in complying with this request. The fact that almost all mothers adhered to the recommendations is encouraging and this practice can potentially be adopted into policy. It was also observed that the recommendation appeared to be acceptable to mothers as they perceived it to be beneficial for their children.

The time of 30 min was chosen since this was assumed to be a reasonable time limit for withholding breastfeeding. Studies show that the half gastric emptying time varies between 47 and 61 min [18-20]. Withholding breastfeeding for an hour before and after may not have been feasible in this setting. Many infants would likely have been offered supplementary food or water and the intervention could inadvertently have interfered with the World Health Organization’s recommendation of exclusive breastfeeding for the first 6 months of life. The 30 min time interval was used in the rotavirus vaccine 116E trials in Delhi [14], which demonstrated good immunogenicity for the vaccine.

At least two other studies are underway to assess the importance of withholding breast milk to improve the immunogenicity of oral vaccines [15,16]. Advising mothers to withhold breast milk around the time of vaccination may be contemplated if there is clear benefit. It is essential that children get the maximum effect from their life-saving vaccines and at the same time it is essential to ensure that the benefits of breastfeeding are not undermined. Clear messages should be developed and tested further before being used in a program setting. It is important that the mothers understand that withholding breastfeeding around the time of vaccination may be required not because there are harmful substances in breast milk but because the beneficial substances may work against the effect of the vaccine.

This study was conducted with a limited population and the investigators did not measure the mothers’ understanding of the breastfeeding recommendations. It is likely that the high compliance seen in this study is an artifact of the study setting for several reasons. Firstly, the recommendations were given by trained study team members with a background in nutrition and skilled in delivering the message. In this setting, it is well known that mothers are more likely to listen to health workers whom they perceive to be of a higher position and qualification: advice given by physicians or nutritionists is more likely to be adhered to. Secondly, the study team members who gave the recommendations also observed the mothers and were present in the same area as the mother–infant pairs. Thirdly, it is possible that the group of mothers who consented to participate in this study were inherent compliers. It is also likely that the mothers’ perceptions of the intervention may have been different in other settings.

The study was conducted in urban resettlement neighborhoods of South Delhi. Though the participants in this study represent an important group, the generalizability of the study is limited since all the participants were from one area of Delhi. Nevertheless, reporting good quality data generated from smaller studies like this is important before considering larger trials in the population.

## Conclusions

In conclusion, mothers in this setting complied with the recommendations given by the study team to withhold breastfeeding or breastfeed half an hour before and after vaccination. It is likely that the mothers perceived the recommendation to have potential benefits to the health of their infants, therefore resulting in the high compliance.

## Abbreviations

IQR: interquartile range; SAS-ERC: Society for Applied Studies, Ethics Review Committee; SD: Standard deviation.

## Competing interests

The authors declare that they have no competing interests.

## Authors’ contributions

All authors contributed to the commencement and design of the manuscript. TRC, NB and TAS were involved in the development of the protocol. TRC, BAW, TAS and NB wrote the manuscript. NG, SSR, MM, RR, AA and FAR were responsible for day-to-day implementation of the study. All authors read and approved the final manuscript.
